# Alpha-Crystallin-Membrane Association Modulated by Phospholipid Acyl Chain Length and Degree of Unsaturation

**DOI:** 10.3390/membranes12050455

**Published:** 2022-04-23

**Authors:** Geraline Trossi-Torres, Raju Timsina, Laxman Mainali

**Affiliations:** 1 Biomolecular Sciences Graduate Program, Boise State University, Boise, ID 83725, USA; geralinetrossito@u.boisestate.edu; 2 Department of Physics, Boise State University, Boise, ID 83725, USA; rajutimsina@boisestate.edu

**Keywords:** α-crystallin, phospholipid acyl chain length, degree of unsaturation, association constant, physical properties of membranes, mobility parameter, maximum splitting, hydrophobicity, barrier hypothesis, cataract

## Abstract

α-crystallin-membrane association increases with age and cataracts, with the primary association site of α-crystallin being phospholipids. However, it is unclear if phospholipids’ acyl chain length and degree of unsaturation influence α-crystallin association. We used the electron paramagnetic resonance approach to investigate the association of α-crystallin with phosphatidylcholine (PC) membranes of different acyl chain lengths and degrees of unsaturation and with and without cholesterol (Chol). The association constant (K_a_) of α-crystallin follows the trends, i.e., K_a_ (14:0–14:0 PC) > K_a_ (18:0–18:1 PC) > K_a_ (18:1–18:1 PC) ≈ K_a_ (16:0–20:4 PC) where the presence of Chol decreases K_a_ for all membranes. With an increase in α-crystallin concentration, the saturated and monounsaturated membranes rapidly become more immobilized near the headgroup regions than the polyunsaturated membranes. Our results directly correlate the mobility and order near the headgroup regions of the membrane with the K_a_, with the less mobile and more ordered membrane having substantially higher K_a_. Furthermore, our results show that the hydrophobicity near the headgroup regions of the membrane increases with the α-crystallin association, indicating that the α-crystallin-membrane association forms the hydrophobic barrier to the transport of polar and ionic molecules, supporting the barrier hypothesis in cataract development.

## 1. Introduction

Cataracts develop in the eye lens, causing hazy, foggy vision and being the leading cause of blindness globally [1]. Aging, which causes age-related changes in the eye lens, is one of the primary causes of cataract development [2,3,4]. Water-soluble crystallin proteins, i.e., α-, β-, and γ-crystallins, account for more than 90% of the lens proteins and function constructively to lens transparency and refractive properties [5,6,7]. α-crystallin accounts for 40% of the lens proteins [6]. The aggregation of crystallin proteins in the lens into the higher molecular weight complexes (HMWCs) [8,9,10,11] accounts for the hazy or foggy vision through the cataractous lens [12,13,14]. Water-soluble α-crystallin slowly decreases with a corresponding increase in its insoluble aggregate with age and onset of cataract [15,16,17]. The aggregation of α-crystallin is a significant factor in cataract formation [18,19]. α-crystallin associates with β- and γ-crystallin forming HMWCs with age [8,9,10,11]. HMWCs are further associated with the fiber cell plasma membrane, followed by light scattering and cataract formation [2,17]. According to a clinical investigation [20], the level of α-crystallin in the eye lens cytoplasm decreases with age and cataract formation, with a corresponding increase in α-crystallin-membrane association, resulting in light scattering and cataract formation. Thus, determining the factors contributing to the α-crystallin-membrane association may be crucial in understanding the early stage of cataract development. It has been reported that the primary association site of α-crystallin in the lens’s membrane is lipids (phospholipids (PLs) and sphingolipids) [21,22,23]. Lipid composition changes dramatically with age, increasing the sphingolipid content [24,25,26,27] and saturation levels of PL acyl chains and decreasing the acyl chain length [24,25,26,27,28]. These changes in lipid composition are observed between the lens cortex and nucleus of clear lenses of different age groups and age-matched cataractous human lenses [29,30,31]. It has been speculated that, with an increase in lipid chain order of membrane with age and cataract [32,33,34], the association of α-crystallin with membrane might increase [35]. It is not clear whether the length of the acyl chain and degree of unsaturation influence α-crystallin-membrane association. Very few studies have been performed earlier to investigate the role of acyl chain length and degree of unsaturation in α-crystallin membrane association, and the results are conflicting. Cobb and Petrash [28] used AlexaFluor350^TM^-conjugated α-crystallin with different synthetic vesicles where vesicles were made of phosphatidylcholine (PC) with different chain length and degree of unsaturation and reported that acyl chain length and degree of unsaturation do not influence the association of α-crystallin to lipid membranes. However, Tang et al. [35] used the fluorophore NBD-PE to investigate the association of α-crystallin with distearoyl-phosphatidylcholine (DSPC), sphingomyelin (SM), and egg-phosphatidylcholine (egg-PC) membranes and found that the α-crystallin-membrane association depends on lipid chain order, with a larger association observed with ordered membranes. 

The association of α-crystallin with membranes alters the physical properties of membranes [2,35], possibly playing a significant role in modulating the integrity of membranes. Borchman and Tang [21] used NBD-PE fluorophore, which resides near the lipid headgroup region, to investigate the physical properties of bovine lens lipid vesicles with the α-crystallin association and discovered that water is excluded from the lipid headgroup regions and headgroup mobility is reduced. It has also been reported that, during aging, lipid headgroup order is modulated in the human lens membrane with the association of α- and β-crystallin [36]. A hypothesis has been made that the association of α-crystallin to lens membrane contributes to nuclear cataract development by obstructing membrane pores and creating a diffusion barrier [16,37,38]. The transport of polar and ionic molecules between fiber cells in the lens is controlled by abundant integral membrane proteins aquaporin-0 (AQP0) and connexins (Cx46 and Cx50) [39,40,41]. It has been reported that ionic imbalance in the lens could cause membrane swelling followed by loss of lens transparency [42,43,44]. The association of α-crystallin to lens membrane may form the diffusion barrier resulting in the disruption of water and ions homeostasis in the lens. However, it is unclear whether changes in the physical properties of the membrane caused by α-crystallin association create a diffusion barrier.

Recently, we have used the electron paramagnetic resonance (EPR) spin-labeling approach to investigate the association of α-crystallin with membranes made of individual lipid [45,46] and two-component lipid mixtures [46,47], where membranes had the same hydrophobic fatty acid core but different headgroups. Our results show that lipid headgroup size and charge, hydrogen bonding between lipid headgroups, and lipid curvature modulate α-crystallin-membrane association and the physical properties of membrane changes with α-crystallin association [2,46,47,48]. Moreover, our results show that, independently of the lipid headgroup, cholesterol (Chol) inhibits α-crystallin-membrane association; however, the level of inhibition was different depending upon the lipid headgroup [2,47,48]. Cobb and Petrash et al. [49] showed that the R116C mutation in αA-crystallin decreased the chaperone-like activity and ability to exchange subunits by 4-fold and increased the αA R116C membrane association capacity by 10-fold, potentially being the cause of congenital cataracts. Srivastava et al. [42] showed that N101D deamidation in αA-crystallin increased association with the mice lens membrane, potentially causing intracellular ionic imbalance and membrane disorganization, successively leading to cortical cataracts. These studies [42,49] are based on the role of α-crystallin mutation and post-translational modification in α-crystallin-membrane association and cataracts. The study reported in this paper investigates the association of α-crystallin with cholesterol (Chol)/phosphatidylcholine (PC) membranes with different chain lengths and degrees of unsaturation (i.e., 1,2-dimyristoyl-sn-glycero-3-phosphocholine (DMPC; 14:0–14:0 PC), 1-stearoyl-2-oleoyl-sn-glycero-3-phosphocholine (SOPC, 18:0–18:1 PC), 1,2-dioleoyl-sn-glycero-3-phosphocholine (DOPC; 18:1–18:1 PC), and 1-palmitoyl-2-arachidonoyl-sn-glycero-3-phosphocholine (PAPC; 16:0–20:4 PC)) and in the presence and absence of 23 mol% Chol. The novelty of this study is that it investigates the role of membrane PLs’ acyl chains by varying the lipid chain length and degree of unsaturation and probes the effect of such variation in the α-crystallin-membrane association. This research also illustrates the role of lipid chain length and degree of unsaturation in modulating the physical properties (maximum splitting, mobility parameter, and hydrophobicity) of membranes with the α-crystallin association. Furthermore, hydrophobicity measured near the membrane surface with α-crystallin association provides new insight into the hydrophobic barrier on the lens membrane surface, supporting the barrier hypothesis in cataract development. 

## 2. Materials and Methods

### 2.1. Materials

DMPC (14:0–14:0 PC), SOPC (18:0–18:1 PC), DOPC (18:1–18:1 PC), PAPC (16:0–20:4 PC), and Cholesterol (Chol) were purchased from Avanti Polar Lipids, Inc. (Alabaster, AL, USA). The cholesterol analog cholestane spin-label (CSL) and bovine eye lens α-crystallin were purchased from Sigma Aldrich (St. Louis, MO, USA), where α-crystallin was used without further purification. Based on the information (αA = 19.8 kDa, αB = 22 kDa, and αA:αB = 3:1), the average molecular weight for the α-crystallin subunit was estimated to be 20.35 kDa. Preparation of membranes, α-crystallin, and α-crystallin membrane association studies was performed in HEPES buffer (10 mM HEPES, 100 mM NaCl, pH = 7.4). 

### 2.2. Sample Preparation for α-Crystallin-Membrane Association Studies

**Figure 1:**
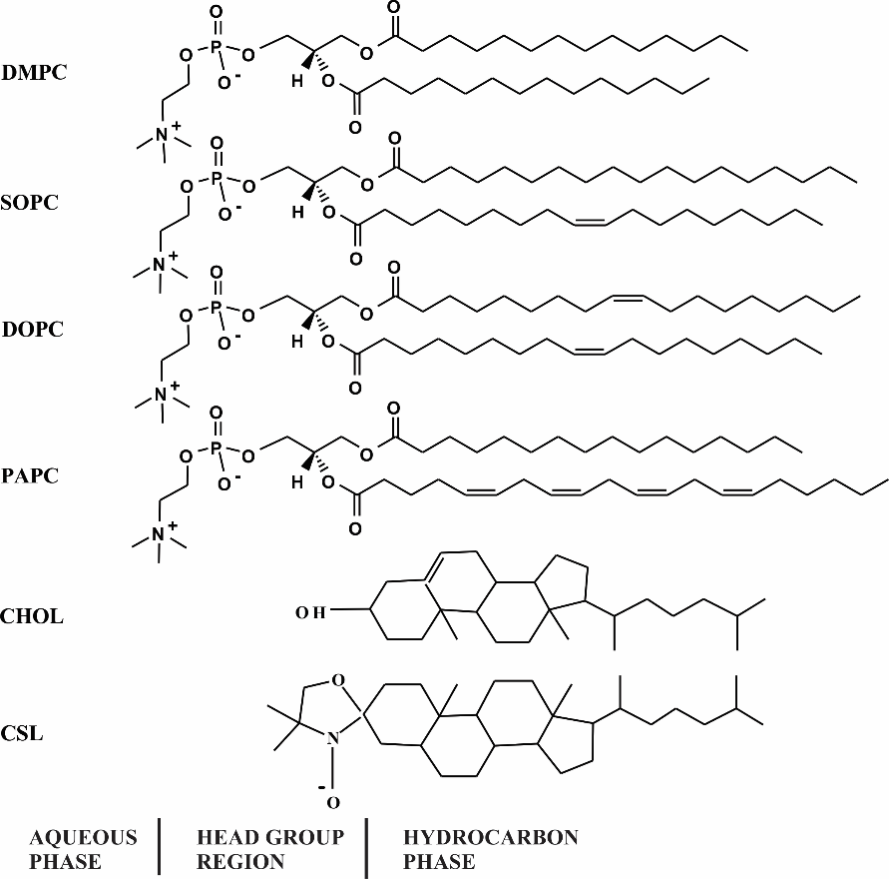
The chemical structures of 14:0–14:0 PC (DMPC), 18:0–18:1 PC (SOPC), 18:1–18:1 PC (DOPC), 16:0–20:4 PC (PAPC), cholesterol (Chol), and cholesterol analogue spin-label (CSL). Approximate locations of each molecule across the lipid bilayer membrane are indicated. The nitroxide moiety of the CSL spin-label resides near the headgroup region.

Phospholipids (PLs) of varying chain lengths and degrees of unsaturation have been chosen to prepare the membrane samples. Figure 1 shows the chemical structure of saturated PL (DMPC) with a short acyl chain length where both chains are saturated, i.e., 14:0–14:0 PC, monounsaturated PL (SOPC) where one acyl chain is saturated and another acyl chain contains one double bond, i.e., 18:0–18:1 PC, polyunsaturated PL (DOPC) where each acyl chain contains one double bond, i.e., 18:1–18:1 PC, and polyunsaturated PL (PAPC) where one acyl chain is saturated and another acyl chain contains four double bonds, i.e., 16:0–20:4 PC. The chemical structure of Chol and CSL with the approximate locations in the PL bilayer membrane is shown in Figure 1.

The PLs (DMPC, SOPC, DOPC, and PAPC), Chol, and CSL in chloroform solutions are mixed with 1 mol% of CSL spin-label maintained in PLs plus Chol solutions. The membrane samples were prepared with Chol/PL mixing ratios of 0 and 0.3. The method for the preparation of small unilamellar vesicles (SUVs) from large multilamellar vesicles (LMVs) using the rapid solvent exchange (RSE) method and probe tip sonication is described in detail in our recent papers [45,46,47]. Briefly, chloroform solutions were dried with a flow of N_2_-gas to the volume of ~75 µL, and then 360 µL of HEPES buffer were added to the chloroform solution to prepare LMVs using the RSE method [47,50,51]. Probe-tip sonicator (Fisher Scientific, Model 550) was used to prepare SUVs by sonicating LMVs ten to fifteen times with 10 s sonication and 15 s in ice for each sonication cycle [46,47]. The PLs plus Chol concentration in the SUVs samples was maintained at 40 mM. Since the autooxidation of the PL membrane depends on the number of double bonds on the acyl chain [52], we prepared the DOPC membrane containing one double bond on each acyl chain and PAPC membranes containing four double bonds on one acyl chain in an N_2_-gas (oxygen-free) environment. It has been reported that the autooxidation of polyunsaturated PAPC membrane is very low for the first 75 h [52]. 

The α-crystallin-membrane association is investigated by incubating α-crystallin with SUVs at 37 °C for 16 h in a shaking incubator (Corning Inc., Corning, NY, USA). The concentration of PLs plus Chol was fixed at 11.4 mM, and the concentration of α-crystallin was varied from 0 to 52.6 μM to monitor the α-crystallin-membrane association. 

### 2.3. EPR Approach for the Investigation of the α-Crystallin-Membrane Association

The α-crystallin membrane samples incubated at 37 °C for 16 h were filled into 0.8 mm i.d. gas-permeable methylpentene polymer (TPX) capillary [48] for EPR measurements at 37 °C. The EPR measurements at −165 °C were performed using a 1.0 mm i.d. gas-permeable methylpentene polymer (TPX) capillary [48] to get a better signal-to-noise ratio. An X-band Bruker ELEXSYS 500 continuous-wave (CW) EPR spectrometer equipped with temperature-controlled accessories was used for the EPR measurements. 

The nitrogen gas flow was used to deoxygenate the samples and maintain the temperature at 37 °C, whereas, for low-temperature measurement, liquid nitrogen was used to maintain the temperature at −165 °C. For 37 °C EPR measurement, CW EPR spectra were accumulated with microwave power and modulation amplitude of 8.0 mW and 1.0 G, respectively. For −165 °C measurement, microwave power of 2.0 mW and modulation amplitude of 2.0 G were used. As described in detail in our recent papers [45,46,47], a change in the EPR signal of the CSL spin-label, located near the headgroup regions of membranes, provides the unique opportunity to investigate the α-crystallin-membrane association. We found no significant change in the EPR signals of CSL in membranes with incubation at 37 °C for 16 h and without incubation (0 h incubation), indicating the stability of membranes. 

The EPR method to estimate the percentage of membrane surface occupied (MSO) by α-crystallin and the association constant (K_a_) is based on the decrease in the peak to peak EPR signal intensity of the low field EPR line with α-crystallin association with membranes and is described in details on our previous works [45,46,47]. The EPR approach to measuring the physical properties of the membranes (maximum splitting, mobility parameter, and hydrophobicity) with an α-crystallin association is explained in our previous studies [45,46,47]. The mobility parameter and maximum splitting are obtained from the EPR signal recorded at 37 °C, and hydrophobicity (2A_z_) is obtained from the EPR signal recorded at −165 °C. The mobility parameter gives information about the mobility (dynamics) change near the headgroup region of membranes with α-crystallin association [45,46,47,48]. The maximum splitting provides information about the order near the headgroup region of membranes with α-crystallin association [45,46,47,48]. The hydrophobicity measured with the CSL spin-label provides information about the surface hydrophobicity near the headgroup regions of the membrane [48], where a decrease in the 2A_z_ value means an increase in hydrophobicity [53,54,55,56,57]. 

### 2.4. Statistics

The statistically significant difference in the maximum percentage of membrane surface occupied (MMSO), K_a_, mobility parameter, maximum splitting, and hydrophobicity is determined using a Student’s *t*-test with *p* ≤ 0.05 as the statistical significance criterion. The MMSO value is the MSO value obtained after achieving saturable binding. All the data are obtained from three independent experiments and expressed with mean ± standard deviation.

## 3. Results and Discussion

### 3.1. MSO by α-Crystallin on Saturated, Monounsaturated, and Polyunsaturated Membranes 

**Figure 2:**
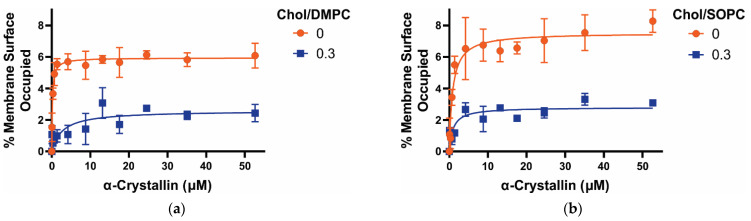

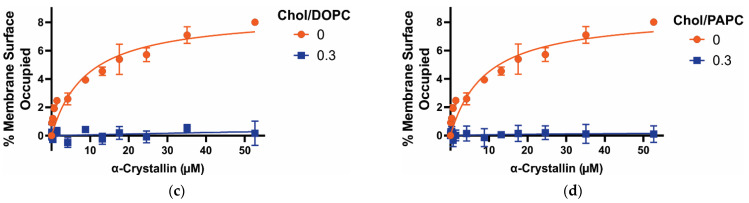
(**a**–**d**) Show the percentage of membrane surface occupied (MSO) plotted as a function of α-crystallin concentration for Chol/DMPC, Chol/SOPC, Chol/DOPC, and Chol/PAPC membranes, respectively, at Chol and PL mixing ratios of 0 and 0.3. A fixed concentration of PL plus Chol (11.4 mM) in the membrane and varied concentration of α-crystallin (0–52.6 μM) was mixed and incubated at 37 °C for 16 h and EPR measurements were taken at 37 °C. The detailed method to calculate the MSO is explained in our previous studies [45,46,47]. The data points are expressed as mean ± standard deviation from three independent experiments and fitted using a one-site ligand binding model (see Equation (2) in [46,47]) in GraphPad Prism (San Diego, CA, USA) to calculate the association constant (K_a_). The rate of increase of MSO with respect to α-crystallin concentration is different for different PC membranes, showing that acyl chain length and degree of unsaturation of PC membranes modulate the α-crystallin-membrane association. Independent of the PC lipid type, Chol decreases the MSO, representing that Chol inhibits the α-crystallin-membrane association.

For all Chol-free membranes (Chol/PC mixing ratio of 0), MSO by α-crystallin increases with an increase in α-crystallin concentration, as shown in Figure 2, representing the increase in the α-crystallin-membrane association. However, the MSO increases rapidly with the rapid saturable association of α-crystallin with DMPC and SOPC membranes compared to DOPC and PAPC membranes. Saturable association of α-crystallin with a lipid membrane was reported previously [21,23,58]. We recently observed the saturable association of α-crystallin with membranes made of individual, two-component, and four-component lipid mixtures [2,45,46,47,48]. All the membranes investigated in this study have the same headgroup but different chain lengths and degrees of acyl chain unsaturation. The different rates to achieve the saturable association of α-crystallin (i.e., MSO by α-crystallin per increase in α-crystallin concentration) with different membranes (Figure 2) suggest that the acyl chain length and degree of unsaturation determine how quickly the saturable association is observed with membranes. As shown in Figure 1, the DMPC membrane has both acyl chains saturated (no double bonds) with 14 carbon atoms in each acyl chain (i.e., 14:0–14:0 PC), the SOPC membrane has one acyl chain saturated and another acyl chain monounsaturated with 18 carbon atoms in each acyl chain (i.e., 18:0–18:1 PC), the DOPC membrane has both acyl chains monounsaturated with 18 carbon atoms in each acyl chain (i.e., 18:1–18:1 PC), and PAPC membrane has one acyl chain saturated with 16 carbon atoms and another acyl chain polyunsaturated with four double bonds with 20 carbon atoms (i.e., 16:0–20:4 PC). Figure 2 shows that the rate to achieve the saturable association of α-crystallin follows the trends: DMPC > SOPC > DOPC ≈ PAPC, suggesting that the membrane with a shorter acyl chain length and a higher degree of saturated chain achieves the binding saturation at a faster rate. The MMSO by α-crystallin follows the trends: SOPC ≈ DOPC > DMPC ≈ PAPC. The MMSO by α-crystallin with DMPC and SOPC membranes are ~6.1% and ~8.3%, respectively, and the difference in these MMSO values is statistically significant with *p* ≤ 0.05. The MMSO by α-crystallin with DOPC and PAPC membranes are ~8% and ~5.9%, respectively, and the difference in these MMSO values is statistically significant with *p* ≤ 0.05. The combined results for DMPC, SOPC, DOPC, and PAPC suggest that how quickly the binding saturation is achieved and the MMSO by α-crystallin is determined by the synergistic effect of both acyl chain length and degree of unsaturation of acyl chains. The MMSO values for Chol-free membranes (Figure 2) reported in this paper are comparable to the MMSO values obtained earlier, i.e., ~2% to ~13%, with membranes made of individual lipid, two-component lipid mixtures, and four-component lipid mixtures using an EPR spin-labeling approach [45,46,47,48]. The MMSO values for Chol-free membranes (Figure 2) are also comparable to the results obtained by Mulders et al. [59], where they labeled α-crystallin with [35S] methionine and incubated with PC vesicles and found that approximately 10% of α-crystallin associated with PC vesicles. We previously investigated the association of α-crystallin with membranes made of individual lipids (i.e., POPC, POPS, and POPE) and two-component lipid mixtures (i.e., SM/POPC, SM/POPS, and SM/POPE), having the same acyl chain length and degree of unsaturation but different lipid headgroups, and found that MMSO by α-crystallin and the rate to achieve the binding saturation is modulated by lipid headgroup’s charge and size, lipid curvature, and hydrogen bonding between lipid headgroups [46]. In this current study, we kept the lipid headgroup fixed and varied acyl length and degree of unsaturation (see Figure 1 for lipid structure) and found that acyl chain length and degree of unsaturation strongly modulate α-crystallin membrane association (Figure 2). Previously, Tang et al. [35] reported that the association of α-crystallin with lipid membrane depends on acyl chain order. However, Cobb and Petrash [28] reported that acyl chain length, unsaturation, and lipid headgroup and type do not influence α-crystallin-membrane association. The earlier infrared spectroscopy study suggests that the lipid headgroup mediates α-crystallin-membrane interactions [60]. 

With Chol/PC mixing ratio of 0.3 (Figure 2), the MSO by α-crystallin sharply decreases close to zero for polyunsaturated membranes (i.e., Chol/DOPC and Chol/PAPC); however, it decreases ~60% for saturated (i.e., DMPC) and monounsaturated (i.e., SOPC) membranes. MMSO values, when compared with and without Chol, show a statistically significant difference with *p* ≤ 0.05 for all PC membranes. These results suggest that Chol decreases the MSO by α-crystallin; however, the decrease level depends on the acyl chain length and degree of unsaturation. We have recently found a similar decrease in MSO by α-crystallin in the presence of Chol for membranes with different headgroups but the same acyl chain length and degree of unsaturation; however, the level of decrease depends upon the lipid headgroup type [47]. Previously, Tang et al. [35] investigated the association of α-crystallin with Chol/distearoyl-phosphatidylcholine (DSPC), Chol/SM, and Chol/egg PC membranes using a fluorescence approach and found that Chol decreases the α-crystallin association with DSPC and SM membranes but increases the α-crystallin association with egg PC membrane. In contrast, Cobb and Petrash [28] used AlexaFluor350TM-conjugated α-crystallin and found no significant association of α-crystallin with and without Chol in PC and SM membranes. The surface hydrophobicity of DMPC, SOPC, DOPC, and PAPC membranes decreases with an addition of Chol (see Section 3.5) irrespective of the acyl chain length and degree of unsaturation. The decrease in surface hydrophobicity with the corresponding decrease in MSO with the addition of Chol suggests that the α-crystallin interacts with the membrane via hydrophobic interaction.

### 3.2. K_a_ of α-Crystallin Association with Saturated, Monounsaturated, and Polyunsaturated Membranes 

**Figure 3:**
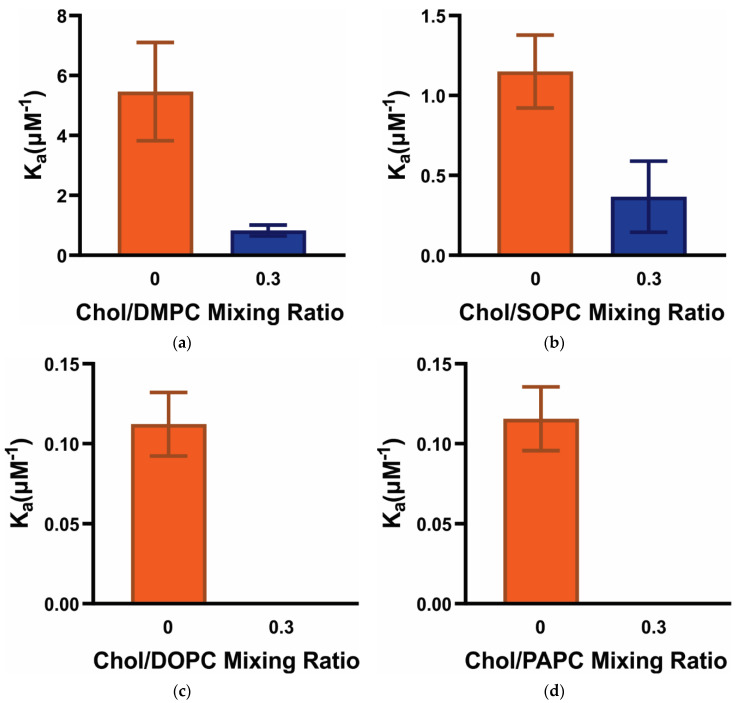
(**a**–**d**) Show the association constant (K_a_) of α-crystallin association with Chol/DMPC, Chol/SOPC, Chol/DOPC, and Chol/PAPC membranes, respectively, at Chol and PL mixing ratios of 0 and 0.3. The K_a_ was calculated by fitting the MSO versus α-crystallin concentration data shown in Figure 2 using a one-site ligand binding model (see Equation (2) in [46,47]) in GraphPad Prism (San Diego, CA) and expressed as mean ± standard deviation from three independent experiments. The K_a_ is different for different PC membranes, representing that acyl chain length and degree of unsaturation strongly modulate the α-crystallin-membrane association. Moreover, the addition of Chol to the PC membranes decreases the K_a_, representing that Chol inhibits the α-crystallin-membrane association. The different levels of decrease in K_a_ for different membranes with the addition of Chol further show that acyl chain length and degree of unsaturation strongly modulate the α-crystallin-membrane association.

To compute the K_a_, the MSO versus α-crystallin concentration data shown in Figure 2 were fitted using a one-site ligand binding model (see Equation (2) in [46,47]) in GraphPad Prism (San Diego, CA, USA). The K_a_ gives the strength of α-crystallin-membrane association. The higher the K_a_, the stronger the α-crystallin-membrane association. If α-crystallin does not associate with the membrane, the MSO and K_a_ values are both 0. The value of K_a_ is determined by how quickly the MSO increases if α-crystallin is associated with the membrane. In other words, K_a_ provides a quantitative estimate of how quickly the MMSO is occupied, and there may be different values of K_a_ for the same MMSO. The K_a_ follows the trends: K_a_ (Chol/DMPC) > K_a_ (Chol/SOPC) > K_a_ (Chol/DOPC) ≈ K_a_ (Chol/PAPC), both for membranes in the presence and absence of Chol, as shown in Figure 3. The K_a_ for the DMPC membrane is about five times higher than the SOPC membrane and about 50 times higher than that of DOPC and PAPC membranes, suggesting that K_a_ is significantly higher for saturated membranes with a shorter acyl chain length. Even the MMSO is similar between DMPC and PAPC, SOPC and DOPC, and Chol/DMPC and Chol/SOPC membranes, the significantly different values of K_a_ for these membranes are attributed to these lipids’ significantly different acyl chain lengths and degrees of unsaturation. Previously, we estimated the K_a_ for POPC (i.e., 16:0–18:1 PC) membrane to be 4.9 ± 2.4 μM^−1^ [45]. In the present study, K_a_ for the SOPC (i.e., 18:0–18:1 PC) membrane is 1.15 ± 0.2 μM^−1^, which is about four times smaller than the K_a_ for the POPC membrane. The only difference between the SOPC and POPC membranes is that one of the acyl chains in the POPC membrane is two carbon atoms shorter than the SOPC membrane. This clearly shows that the shorter the acyl chain length, the higher is the K_a_ of α-crystallin-membrane association. The K_a_ for SOPC and DOPC membranes are 1.15 ± 0.22 μM^−1^ and 0.11 ± 0.02 μM^−1^, respectively. The SOPC and DOPC membranes have 18 carbon atoms in their acyl chains. However, the SOPC membrane has only one acyl chain monounsaturated (i.e., 18:0–18:1 PC), whereas the DOPC membrane has both the acyl chains monounsaturated (i.e., 18:1–18:1 PC). Approximately ten times larger K_a_ for the SOPC membrane than the DOPC membrane clearly shows that the increase in the number of double bonds in the acyl chains (i.e., increase in the level of unsaturation) significantly decreases the K_a_, playing a critical role in the α-crystallin-membrane association. The K_a_ values for DOPC and PAPC membranes and Chol/DOPC and Chol/PAPC membranes are similar with no statistically significant difference with a *p*-value ≤ 0.05. Other than these membranes, the K_a_ values among different membranes and within the same membrane with different Chol content have a statistically significant difference with a *p*-value ≤ 0.05. The PAPC membrane has one acyl chain saturated, and another acyl chain with 20 carbon atoms is polyunsaturated with four double bonds (i.e., 16:0–20:4 PC). The similar K_a_ values for the DOPC and PAPC membranes suggest the synergistic effects of unsaturation of acyl chain, level of polyunsaturation (number of double bonds on acyl chain), and length of acyl chains on α-crystallin-membrane association. Our previous K_a_ measurements for membranes having the same acyl chain length and degree of unsaturation, but different headgroups (i.e., POPC, POPS, POPE, SM/POPC, SM/POPS, and SM/POPE membranes) show K_a_ differs significantly for membranes due to the difference in their headgroups [45,46]. The study reported in this paper suggests that the acyl chain length and degree of acyl chain unsaturation modulate α-crystallin-membrane association. Lipid composition in the human lens membrane changes significantly with age, increasing the saturation of the acyl chains (i.e., decreasing the double bonds) and decreasing the length of the lipid acyl chain [24,25,26,27,28]. The data presented in this study show that shorter acyl chain length and a higher degree of acyl chain saturation significantly increase the K_a_ of α-crystallin-membrane association. This may be one of the reasons why the association of α-crystallin with the lens membrane increases with age [2,12,15,16,20,38]. The estimated K_a_ values (Figure 3) are comparable to the K_a_ values obtained for the membranes made of individual lipid [45,46], two-component lipid mixtures [46], and four-component lipid mixtures [48]. Moreover, the K_a_ values reported in this study are comparable to the K_a_ value of 7.69 μM^−1^ reported earlier by Mulders et al. [59] for the α-crystallin association with alkali-washed lens plasma membranes.

Figure 3 shows that, independently of the acyl chain length and degree of unsaturation, Chol inhibits the association of α-crystallin with Chol/DMPC, Chol/SOPC, Chol/DOPC, and Chol/PAPC membranes; however, the level of inhibition is different for different membranes. In the Chol/DMPC membrane, K_a_ decreases by a factor of about six times when compared to K_a_ of the Chol-free DMPC membrane; however, in the case of the Chol/SOPC membrane, K_a_ decreases by a factor of about three times when compared to Chol-free SOPC membrane (Figure 3). The addition of Chol separates the headgroups of the membrane, increasing the water accessibility (decreasing hydrophobicity) near the headgroup regions, ultimately reducing the strength (K_a_) of the likely hydrophobic α-crystallin-membrane association. The higher decrease in K_a_ values for Chol/DMPC membrane compared to Chol/SOPC membrane with the addition of Chol can be explained based on the corresponding decrease in the surface hydrophobicity of membranes. Our recent study investigating the association of α-crystallin with models of human, porcine, and mouse lens-lipid membranes shows that the surface hydrophobicity of membranes and K_a_ decrease with increased Chol content, suggesting the hydrophobic interaction of α-crystallin to the model lens-lipid membranes [48]. With the addition of Chol, the surface hydrophobicity of the DMPC membrane decreases significantly from its initial value compared to the SOPC membrane (see Section 3.5), which may be why the K_a_ for the Chol/DMPC membrane decreases significantly compared to the Chol/SOPC membrane. For Chol/DOPC and Chol/PAPC membranes, K_a_ decreases significantly close to 0 with the addition of Chol, like MSO (Figure 2), implying no significant association of α-crystallin with these membranes. Our results show that Chol inhibits α-crystallin membrane association; however, the synergistic effect of lipid acyl chain length and degree of unsaturation of membranes strongly modulate the level of inhibition. Previously, we investigated POPC and POPS membranes with the same acyl chains and degree of unsaturation but with different headgroups [45,46]. 50 mol% Chol completely inhibits α-crystallin association with the POPC membrane, but 60 mol% Chol does not completely inhibit α-crystallin association with the POPS membrane, suggesting that the lipid headgroups modulate the level of inhibition of α-crystallin association with membranes [45,46]. The combined results of our previous studies [45,46] and the study reported in this paper suggest that the acyl chain length, degree of acyl chain unsaturation, and lipid headgroups modulate α-crystallin-membrane association.

### 3.3. Mobility Parameter of Saturated, Monounsaturated, and Polyunsaturated Membranes with the α-Crystallin Association

**Figure 4:**
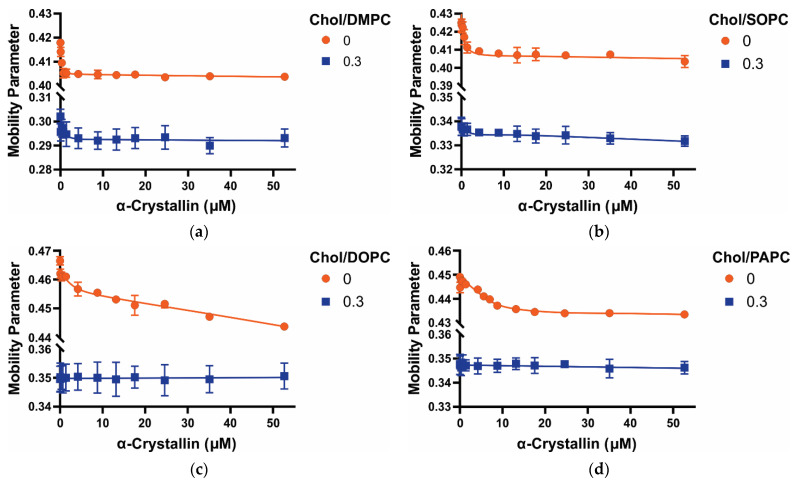
(**a**–**d**) Show the profiles of the mobility parameter (h_+_/h_0_) plotted as a function of α-crystallin concentration for Chol/DMPC, Chol/SOPC, Chol/DOPC, and Chol/PAPC membranes, respectively, at Chol and PL mixing ratios of 0 and 0.3. Our previous studies [45,46,47] explain a detailed method to calculate the mobility parameter (h_+_/h_0_). The data points are expressed as mean ± standard deviation from three independent experiments, and the solid lines serve as the visual guides. The mobility parameters are different for different PC membranes, showing that the mobility near the headgroup regions of membranes depends on the acyl chain length and degree of unsaturation of membranes. For all Chol-free membranes, the mobility parameter decreases with an increase in α-crystallin concentration, representing that these membranes become more immobilized near the headgroup region with the α-crystallin association. The addition of Chol decreases the mobility parameter of membranes, representing that these membranes regions near the headgroup become more immobilized with increased Chol content. However, the decrease in mobility parameter with increased α-crystallin concentration is less pronounced with the addition of Chol in the membranes, showing that Chol inhibits α-crystallin-membrane association. With increased α-crystallin concentration, mobility parameters of Chol/DMPC and Chol/DOPC membranes decrease, unlike for the Chol/DOPC and Chol/PAPC membranes, further showing that the acyl chain length and degree of unsaturation strongly modulate the mobility near the headgroup regions of membranes.

The mobility parameter obtained from the CSL spin-label near the membrane surface gives information about the orientational and rotational mobility near the headgroup regions of the membrane [45,46,47,53,56,61]. In the absence of Chol and α-crystallin, the mobility parameter of membranes follows the trends: DOPC > PAPC > SOPC > DMPC, as shown in Figure 4, indicating that dynamics near the headgroup regions of membranes decrease with the decrease in the degree of unsaturation of acyl chains (i.e., the decrease in the double bonds in acyl chain), with the degree of unsaturation of both acyl chains contributing to the mobility parameter. For Chol-free DMPC, SOPC, DOPC, and PAPC membranes, the mobility parameter values are statistically significant with a *p*-value ≤ 0.05. Interestingly, the trends of K_a_ of α-crystallin association with membranes (Figure 3) are the same as the reverse trends of the mobility parameter of membranes (Figure 4). The K_a_ is significantly high with significantly low mobility near the headgroup regions of saturated (DMPC) and monounsaturated (SOPC) membranes, suggesting that less mobile membranes have significantly higher K_a_. The mobility parameter of the DOPC membrane is greater than the PAPC membrane, suggesting that one double bond in each acyl chain of the DOPC membrane effectively increases the mobility parameter than four double bonds in only one acyl chain of the PAPC membrane. For all Chol-free membranes (Figure 4), the mobility parameter decreases with an increase in α-crystallin concentration, indicating that the membrane regions near the headgroup become more immobilized with the α-crystallin-membrane association. It is clear from Figure 4 that the acyl chain length and degree of unsaturation of membranes determine how fast the mobility parameter decrease with α-crystallin concentration. For the DMPC membrane (Figure 4a) with both acyl chains saturated and with shorter chain length, the mobility parameter decreases sharply with α-crystallin concentration, indicating that the membrane rapidly becomes more immobilized near the headgroup regions. However, for the SOPC membrane (Figure 4b) with longer acyl chains with one chain monounsaturated and other chain saturated, the mobility parameter decreases sharply but not as sharply as in the DMPC membrane. In polyunsaturated DOPC and PAPC membranes (Figure 4c,d), the mobility parameter decreases slowly with an increase in α-crystallin concentration, indicating that the membranes slowly become more immobilized near the headgroup regions with an increase in α-crystallin concentration. All the membranes investigated here have the same lipid headgroup but varying acyl chain length and degree of unsaturation. The profiles of mobility parameter presented in Figure 4 clearly show that the acyl chain length and degree of unsaturation strongly modulate membrane dynamics near the head group regions with and without α-crystallin association. Previously, we investigated membranes with the same acyl chain length and degree of unsaturation but different headgroups and found that the lipid headgroup strongly modulates the mobility parameter near the headgroup regions of membranes with and without α-crystallin association [45,46].

With the addition of the Chol, independently of the acyl chain length and degree of unsaturation, the mobility parameter decreases significantly for all membranes (Figure 4), indicating that the regions of the membranes near the headgroup become more immobilized in the presence of Chol. In the presence of Chol and absence of α-crystallin, the mobility parameter follows the trends: Chol/DOPC ≈ Chol/PAPC > Chol/SOPC > Chol/DMPC, indicating that dynamics near the headgroup regions in the presence of Chol is smallest for the saturated membrane and largest for the polyunsaturated membrane. Even in the presence of Chol, membranes with shorter and saturated acyl chains (Chol/DMPC) have lower mobility parameters compared to a membrane having longer acyl chains with one chain being monounsaturated (Chol/SOPC). The mobility parameters have a statistically significant difference with a *p*-value ≤ 0.05 for all the Chol-containing membranes except for the Chol/DOPC and Chol/PAPC membranes. Interestingly, the trends of K_a_ for Chol-containing membranes (Figure 3) are the same as the reverse trends of the mobility parameter (Figure 4), indicating that the less mobile membranes near the headgroup regions have high K_a_, like Chol-free membranes discussed above. No significant difference between the mobility parameter of Chol/PAPC and Chol/DOPC membranes was observed, indicating that DOPC with one double bond on each acyl chain and PAPC with one acyl chain saturated and another acyl chain with four double bonds have a similar effect on the dynamics near the headgroup regions of membranes with Chol. Mobility parameters decrease with an increase in α-crystallin concentration for Chol/DMPC and Chol/SOPC membranes (Figure 4a,b), unlike for Chol/DOPC and Chol/PAPC membranes (Figure 4c,d). These results show that Chol modulates the mobility parameter of saturated, monounsaturated, and polyunsaturated membranes with increased α-crystallin concentration differently. The decrease in the mobility parameter of Chol/DMPC and Chol/SOPC membranes with the α-crystallin association is not as pronounced as for the same membranes without Chol, indicating that Chol decreases the capacity of α-crystallin to decrease the mobility parameter. As expected, the mobility parameter of Chol/DOPC and Chol/PAPC membranes do not significantly change with an increase in α-crystallin concentration (Figure 4c,d). This is because there is no association or minimal association of α-crystallin with these membranes, as seen from the MSO versus α-crystallin concentration data shown in Figure 2c,d. Previously, we investigated the α-crystallin association with Chol/POPC, Chol/POPS, and Chol/POPE membranes with the same acyl chain length and degree of unsaturation but different headgroups and found that the effect of Chol on modulating the mobility parameter of membranes significantly depends on the lipid headgroup type [47]. The results reported in Figure 4 suggest the synergistic effect of acyl chain length and degree of unsaturation and Chol modulate the membrane dynamics near the headgroup regions with the α-crystallin association. The association of α-crystallin with vesicles made of bovine lens-lipid was investigated previously using fluorophore NBD-PE, which resides near the membrane surface, and found that headgroup mobility of the membrane decreases with the α-crystallin association [21], as reported in this study.

### 3.4. Maximum Splitting of Saturated, Monounsaturated, and Polyunsaturated Membranes with the α-Crystallin Association

**Figure 5:**
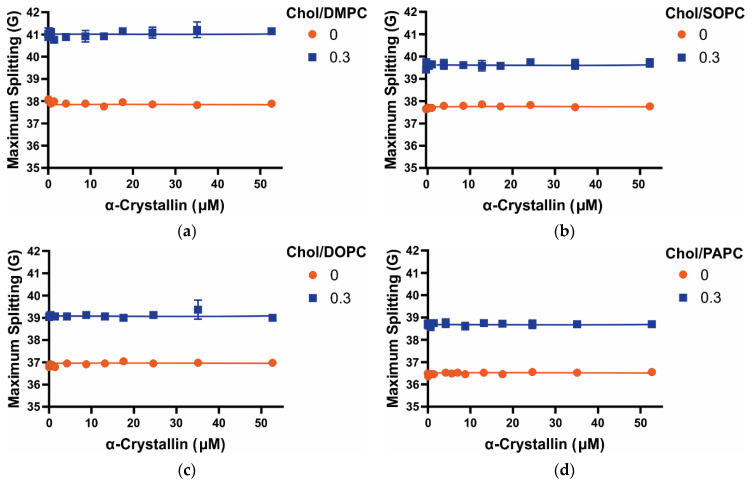
(**a**–**d**) Show the profiles of the maximum splitting plotted as a function of α-crystallin concentration for Chol/DMPC, Chol/SOPC, Chol/DOPC, and Chol/PAPC membranes, respectively, at Chol and PL mixing ratios of 0 and 0.3. Our previous studies [45,46,47] explain a detailed method to calculate the maximum splitting. The data points are expressed as mean ± standard deviation from three independent experiments, and the solid lines serve as the visual guides. The maximum splitting values are different for different PC membranes, indicating that the order of the membranes near the headgroup regions depends on the acyl chain length and degree of unsaturation of membranes. The trends of the maximum splitting are the same as the trends of the K_a_ (see Figure 3), showing the direct correlation between maximum splitting and the K_a_. For all the Chol-free and Chol-containing PC membranes, the maximum splitting does not significantly change with an increase in α-crystallin concentration, representing that the order near the headgroup regions of these membranes does not significantly change with or without α-crystallin association. The addition of Chol increases the maximum splitting of membranes, representing that these membranes become more ordered near the headgroup regions with increased Chol content.

The maximum splitting measured from the EPR spectra of the CSL spin-label located near the membrane surface gives the amplitude of wobbling motion of the long axis of the CSL molecule in the membrane [46,47,53,56,62]. The maximum splitting is related to the order parameter, and the higher value of maximum splitting measured with the CSL spin-label in the membrane indicates more membrane order near the headgroup region and vice-versa [46,47,53,56,62]. In the absence of Chol and α-crystallin, maximum splitting followed the trends: DMPC > SOPC > DOPC > PAPC, indicating that the saturated membrane with shorter acyl chains has maximum membrane order near the headgroup regions. The significant decrease in the membrane order near the headgroup regions of polyunsaturated DOPC and PAPC membranes than the saturated DMPC and monounsaturated SOPC membranes suggests a higher degree of acyl chain unsaturation significantly decreases the membrane order near the headgroup region (Figure 5). Interestingly, the trends of K_a_ of α-crystallin association with the membranes (Figure 3) and the trends of the maximum splitting of the membranes (Figure 5) are the same. With the significant decrease in the membrane order near the headgroup regions of polyunsaturated (DOPC and PAPC) membranes, the K_a_ also decreases significantly, showing the direct correlation between the membrane order near the headgroup region and the K_a_. There is a slight decrease in order near the headgroup region of the PAPC membrane than the DOPC membrane; however, there is no significant difference between the K_a_ values of these membranes. For all the Chol-free membranes with an increase in α-crystallin concentration, the maximum splitting of membranes does not change significantly (Figure 5), indicating that membranes order near the headgroup does not change significantly with the α-crystallin association. In our previous studies, with an increase in α-crystallin concentration, except for the SM and SM/POPE membranes [46], we observed no significant changes in the maximum splitting of membranes made of individual lipids and two-component lipid mixtures [45,46], Chol-containing lipid [47], and Chol-containing four-component lipid mixtures [48].

With the addition of Chol, maximum splitting increases for all the membranes irrespective of the acyl chain length and degree of unsaturation (Figure 5), indicating that the Chol increases the membrane order near the headgroup regions. In the presence of Chol, maximum splitting follows the trends: Chol/DMPC > Chol/SOPC > Chol/DOPC > Chol/PAPC like Chol-free membrane; however, maximum splitting is significantly higher (i.e., larger membrane order) for saturated (Chol/DMPC) membrane compared to monounsaturated (Chol/SOPC) and polyunsaturated (Chol/DOPC and Chol/PAPC) membranes. The maximum splitting values have a statistically significant difference with a *p*-value ≤ 0.05 for all the Chol/DMPC, Chol/SOPC, Chol/DOPC, and Chol/PAPC membranes at Chol and PL mixing ratios of 0 and 0.3. Interestingly, the trends of K_a_ for Chol-containing membranes (Figure 3) are the same as the trends of the maximum splitting (Figure 5), indicating that the membranes with high order near the headgroup regions have high K_a_, like Chol-free membranes discussed above. For all the Chol-containing membranes, the maximum splitting does not significantly change with an increase in α-crystallin concentration (Figure 5), indicating that membrane order near the headgroup regions does not significantly change with an increase in α-crystallin concentration.

Human lens lipid composition changes significantly with age, increasing the saturation of acyl chains (i.e., decreasing the double bonds) and a decrease in chain length [24,25,26,27,28]. This may be why the association of α-crystallin with the lens membrane increases with age [2]. It has also been speculated that the lipid order of the membrane increases with age and cataracts [32,33], resulting in a proportional increase in α-crystallin-membrane association [35].

### 3.5. Surface Hydrophobicity of Saturated, Monounsaturated, and Polyunsaturated Membranes with the α-Crystallin Association

**Figure 6:**
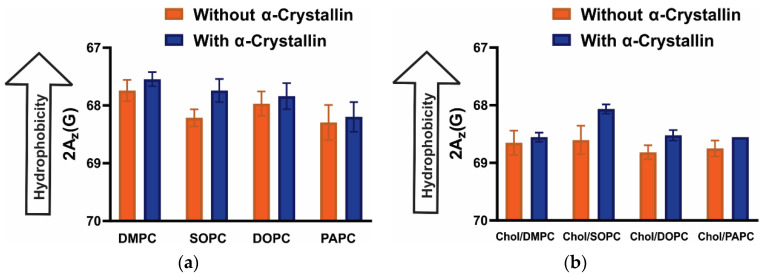
(**a**) Shows the profiles of hydrophobicity (2A_z_) with and without α-crystallin for DMPC, SOPC, DOPC, and PAPC membranes; (**b**) shows the profiles of hydrophobicity with and without α-crystallin for Chol/DMPC, Chol/SOPC, Chol/DOPC, and Chol/PAPC membranes with Chol and PL mixing ratio of 0.3. Membrane and α-crystallin membrane samples incubated at 37 °C for 16 h, which are used to measure the MSO shown in Figure 2, are used for hydrophobicity measurements at −165 °C. The 52.6 µM of α-crystallin is used for all the hydrophobicity measurements presented for the α-crystallin membrane samples in Figure 6. Our previous study [48] explains a detailed method to calculate hydrophobicity. The data points are expressed as mean ± standard deviation from three independent experiments. Without Chol (Figure 6a), the hydrophobicity for all membranes slightly increases with α-crystallin, representing that the α-crystallin-membrane association slightly increases hydrophobicity (decreases polarity) near the headgroup regions of membranes. Interestingly with Chol (Figure 6b), the hydrophobicity for all membranes slightly increases with α-crystallin; even no or minimal association of α-crystallin is observed with Chol/DOPC and Chol/PAPC membranes. This suggests that the α-crystallin close to these membrane surfaces expels the water molecules around the headgroup regions, increasing hydrophobicity (decreasing polarity).

The z-component of the hyperfine interaction tensor (A_z_) measured from the EPR spectra of frozen (approximately −165 °C) samples gives the measure of the hydrophobicity [48,53,54,55,56,57]. The 2A_z_ value measured from the frozen membrane samples using the CSL spin-label gives the measure of hydrophobicity near the headgroup region of the membrane [48,55,56,57]. The decrease in the 2A_z_ value of the CSL spin-label in the membrane means an increase in hydrophobicity (i.e., decrease in polarity) around the nitroxide moiety of CSL [48,53,54,55,56,57]. The nitroxide moiety of CLS resides near the headgroup region of the membrane, as shown in Figure 1. The hydrophobicity near the headgroup regions of membranes slightly increases with the α-crystallin association, as shown in Figure 6a. Previously, we have suggested the hydrophobic interaction of α-crystallin with membranes [46,47,48], with hydrophobic residues exposed on the surface of α-crystallin associating with membranes [46,47]. It is likely that the α-crystallin, with exposed hydrophobic residues on its surface, associates with the membrane expelling out the water molecules around the polar headgroup regions of the membrane, slightly increasing the hydrophobicity (decreasing the polarity) near the headgroup regions of the membrane. The increase in hydrophobicity is more pronounced for α-crystallin association with the SOPC membrane than with other membranes (Figure 6a), suggesting that acyl chain length and degree of unsaturation modulate hydrophobicity near the headgroup regions of membranes. The hydrophobicity values with and without α-crystallin association with the SOPC membrane have a statistical significance difference with a *p*-value ≤ 0.05. Other than the SOPC membrane, there is a slight increase in hydrophobicity with an increase in α-crystallin concentration for other Chol-free membranes; however, the hydrophobicity values have no statistically significant difference with a *p*-value ≤ 0.05.

**Figure 7:**
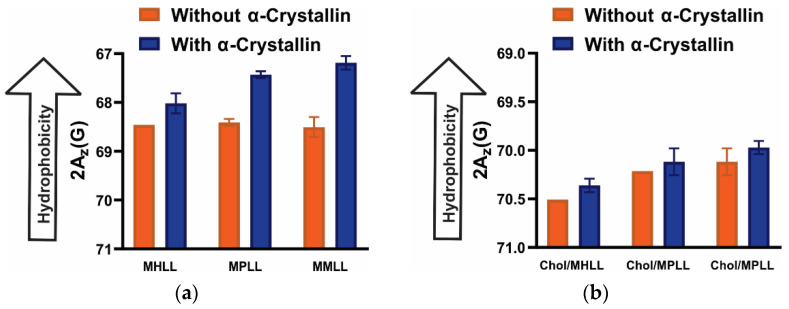
(**a**) Shows the profiles of hydrophobicity (2A_z_) with and without α-crystallin for MHLL, MPLL, and MMLL membranes; (**b**) shows the profiles of hydrophobicity with and without α-crystallin for Chol/MHLL, Chol/MPLL, and Chol/MMLL membranes with a Chol/MHLL mixing ratio of 1.5 and Chol/MPLL and Chol/MMLL mixing ratio of 1. Membrane and α-crystallin-membrane samples incubated at 37 °C for 16 h, which are used to measure the MSO shown in Figure 3 of [48], are used for hydrophobicity measurements at −165 °C. The 52.6 µM of α-crystallin is used for all the hydrophobicity measurements presented for the α-crystallin membrane samples in Figure 7. Our previous study [48] explains a detailed method to calculate hydrophobicity. The data points are expressed as mean ± standard deviation from three independent experiments. Without Chol (Figure 7a), the hydrophobicity for all membranes increases with α-crystallin, representing that the α-crystallin-membrane association increases hydrophobicity (decreases polarity) near the headgroup regions of these membranes. Interestingly, the hydrophobicity for Chol/MHLL, Chol/MPLL, and Chol/MHLL membranes slightly increases with α-crystallin, even no or minimal association of α-crystallin is observed for these membranes (see Figure 3 of ref [48]), suggesting that the α-crystallin close to the membrane surface expels the water molecules around the headgroup regions of membranes, increasing hydrophobicity (decreasing polarity). Data for MHLL, MPLL, and MMLL membranes with and without Chol were redrawn with permission from [48], Copyright 2022, with permission from Taylor & Francis.

Chol is a major lens membrane component [63], the addition of which in PL membranes decreased hydrophobicity (Figure 6b) compared to the corresponding Chol-free membranes (Figure 6a), indicating that Chol decreases the hydrophobicity near the headgroup regions of membranes independently of the PL acyl chain length and degree of unsaturation. Chol separates the PL’s headgroups allowing water penetration in the region, decreasing hydrophobicity [57]. Our previous study [48] also shows that the addition of Chol decreases hydrophobicity near the headgroup regions of PL membranes. In addition, Chol decreases the mobility parameter, as shown in Figure 4 and our previous studies [47,48], and increases the maximum splitting, as shown in Figure 5 and our previous studies [47,48], representing the fact that membrane regions near the headgroup becomes less mobile and more ordered with the addition of Chol. It has also been reported that Chol modulates nuclear and cortical lens lipids’ structural order [33]. Interestingly, the hydrophobicity near the headgroup regions of the membrane decreases with the addition of Chol (Figure 6a,b), accompanied by the decrease in the MSO (Figure 2) and K_a_ (Figure 3), suggesting the hydrophobic interaction of α-crystallin with the membrane. Recently, we investigated the association of α-crystallin with Chol/model of human lens-lipid (Chol/MHLL), Chol/model of porcine lens-lipid (Chol/MPLL), and Chol/model of mouse lens-lipid (Chol/MMLL) membranes and discovered a decrease in MSO, K_a_, and hydrophobicity with increasing Chol content, implying that α-crystallin interacts with these membranes hydrophobically [48]. Research performed in other laboratories using different approaches (i.e., fluorescence [64], resonance energy transfer [14], and heat treatment [65]) suggested that α-crystallin interacts with the membrane via hydrophobic interaction. Like Chol-free membranes discussed above in Figure 6a, hydrophobicity near the headgroup region of Chol-containing membranes slightly increases with the α-crystallin (Figure 6b), forming the hydrophobic barrier near the membrane surface. Except for the Chol/SOPC membrane, the hydrophobicity values for the Chol/DMPC, Chol/DOPC, and Chol/PAPC membranes with and without α-crystallin have no statistically significant difference with a *p*-value ≤ 0.05. Interestingly, the hydrophobicity of Chol/DOPC and Chol/PAPC membranes slightly increases in the presence of α-crystallin, despite no or minimal association of α-crystallin with these membranes (see Figure 2c,d). We speculate that, even if α-crystallin has no or minimal association with the membrane, water molecules around the headgroup regions of the membrane are likely to be expelled as α-crystallin with exposed hydrophobic sites approaches the membrane, increasing hydrophobicity around the headgroup regions. A similar slight increase in hydrophobicity near the headgroup regions of the Chol/MHLL, Chol/MPLL, and Chol/MMLL membranes was observed (see Figure 7), despite no or minimal association of α-crystallin with these membranes [48].

Figure 7a shows that the α-crystallin association with the MHLL, MPLL, and MMLL membranes increases the hydrophobicity near the headgroup regions of these membranes forming the hydrophobic barrier; however, the increase in hydrophobicity depends upon the lipid composition. Hydrophobicity data shown in Figure 7 without α-crystallin were taken from our recent study [48]. The increase in hydrophobicity with α-crystallin association followed the trends: MMLL > MPLL > MHLL. The MMLL, MPLL, and MHLL membranes are made of four major lipids of the eye lens membrane (i.e., SM, POPC, POPS, and POPE). The POPC content in MMLL, MPLL, and MHLL membranes are 46%, 35%, and 11%, respectively and SM content in MMLL, MPLL, and MHLL membranes are 15%, 29%, and 66%, respectively [48]. These data suggest that the increase in hydrophobicity with α-crystallin association increases with an increase in POPC content and decrease in SM content, suggesting that the lipid composition strongly modulates hydrophobicity near the headgroup region of the membrane with the α-crystallin association. For MHLL, MPLL, and MMLL membranes, the hydrophobicity values with and without α-crystallin have a statistically significant difference with a *p*-value ≤ 0.05. Surprisingly, even with high Chol content (Chol/MHLL mixing ratio of 1.5, and Chol/MPLL and Chol/MMLL mixing ratio of 1), we see a slight increase in hydrophobicity with an increase in α-crystallin concentration (Figure 7b); however, the increase is not as pronounced as for Chol-free MHLL, MPLL, and MMLL membranes (Figure 7a). Except for the Chol/MHLL membrane, the hydrophobicity values for the Chol/MPLL and Chol/MMLL membranes with and without α-crystallin have no statistically significant difference with a *p*-value ≤ 0.05. Previously, we found MSO by α-crystallin to be zero or close to zero for Chol/MPLL and Chol/MPLL membranes with a mixing ratio of 1 and Chol/MHLL membrane with a mixing ratio of 1.5, indicating no association or minimal association of α-crystallin with these membranes [48]. As explained above, we speculate that, even if α-crystallin has no or minimal association with these membranes, water molecules around the headgroup regions of membranes are likely to be expelled as α-crystallin with exposed hydrophobic sites approaches membranes, increasing hydrophobicity around the headgroup regions of these membranes.

The increase in hydrophobicity near the headgroup regions of membranes with α-crystallin association forms the hydrophobic barrier to the transport of polar and ionic molecules. It has been proposed earlier that the α-crystallin associated with lens membrane in the barrier regions after middle age may result in occluding membrane pores and creating an oxidative condition in the lens followed by the development of nuclear cataracts [16,37,38]. Although the study conducted here does not include membrane proteins, the results indicating the increase in hydrophobicity (i.e., hydrophobic barrier) near the headgroup region of the membrane with α-crystallin association support the barrier hypothesis that α-crystallin association to lens membrane block the transport of metabolites and ions that leads to developing nuclear cataract [16,37,38].

## 4. Conclusions

We investigated the association of α-crystallin with Chol/PC membranes of varying chain length and degree of unsaturation and measured the MSO, K_a_, and the physical properties (mobility parameter, maximum splitting, and hydrophobicity) of membranes with the α-crystallin association. The MSO increases with an increase in α-crystallin concentration, indicating an increase in the α-crystallin-membrane association. However, the MSO increases rapidly for saturated and monounsaturated membranes than the polyunsaturated membranes. Independently of the acyl chain length and degree of unsaturation, Chol decreases the MSO; however, the decrease in MSO is more significant for polyunsaturated membranes than saturated and monounsaturated membranes. For all membranes with and without Chol, K_a_ follows the trends, i.e., K_a_ (14:0–14:0 PC) > K_a_ (18:0–18:1 PC) > K_a_ (18:1–18:1 PC) ≈ K_a_ (16:0–20:4 PC), with the saturated and monounsaturated membranes having significantly higher K_a_ than the polyunsaturated membranes. The mobility parameter of polyunsaturated membranes is significantly higher than the saturated and monounsaturated membranes, representing that the polyunsaturated membranes are more mobile near the headgroup regions than the saturated and monounsaturated membranes. With increased α-crystallin concentration, the mobility parameter decreases sharply for saturated and monounsaturated membranes than polyunsaturated membranes, indicating that saturated and monounsaturated membranes rapidly become more immobilized near the headgroup regions than polyunsaturated membranes. For all membranes with and without Chol, the maximum splitting does not significantly change with an increase in α-crystallin concentrations. However, maximum splitting is significantly higher for saturated and monounsaturated membranes than polyunsaturated membranes, indicating that saturated and monounsaturated membranes are more ordered near the headgroup regions than polyunsaturated membranes. Our results directly correlate the mobility and order near the headgroup regions of membranes with the K_a_, with the less mobile and more ordered membrane having substantially higher K_a_. Furthermore, our results show that the α-crystallin-membrane association increases the hydrophobicity near the headgroup regions of the membrane creating the hydrophobic barrier. The hydrophobic barrier occludes the membrane pores obstructing the transport of polar and ionic molecules, creating an oxidative condition in the lens followed by the nuclear cataract development [16,37,38]. Although the membranes used in this study do not contain membrane proteins as in the in-vivo condition, our study supports the barrier hypothesis that the association of α-crystallin with the lens membrane blocks the transport of ions and metabolites, leading to the development of nuclear cataract [16,37,38]. The study reported in this paper shows that the lipid type (acyl chain length and degree of unsaturation) modulates the α-crystallin-membrane association and physical properties (hydrophobicity, mobility parameter, and maximum splitting) of the membrane. Our previous studies [45,46] show that the lipid headgroups modulate the α-crystallin-membrane association and physical properties of the membrane. The combined results of our previous studies [45,46] and the study reported in this paper suggest that the acyl chain length, degree of acyl chain unsaturation, and lipid headgroups modulate α-crystallin-membrane association and the physical properties of the membrane. Lipids (PLs and sphingolipids) are the primary association sites of α-crystallin [21,22,23]. The lens lipid composition changes significantly with age, increasing the saturation of acyl chains and decreasing the chain length [24,25,26,27,28]. Moreover, the α-crystallin-membrane association increases with age and cataract formation [2,20,35]. The findings reported in this paper provide profound insights for understating the role of acyl chain length and degree of unsaturation in modulating the α-crystallin-membrane association and the membrane’s physical properties.

## Data Availability

On reasonable request, the data will be available from the corresponding author.
